# HIV-associated adipose redistribution syndrome (HARS): definition, epidemiology and clinical impact

**DOI:** 10.1186/1742-6405-4-16

**Published:** 2007-07-16

**Authors:** Kenneth Lichtenstein, Ashok Balasubramanyam, Rajagopal Sekhar, Eric Freedland

**Affiliations:** 1 University of Colorado Infectious Disease Group Practice, Denver, CO, USA; 2 Translational Metabolism Unit, Division of Diabetes, Endocrinology and Metabolism, Baylor College of Medicine, Houston, TX, USA; 3 EMD Serono, Inc., Rockland, MA, USA

## Abstract

A segment of the HIV infected population develops abnormal and excessive accumulation of adipose tissue in the trunk, including accumulation of visceral (deep abdominal) adipose tissue. This condition, known as HIV-related adipose redistribution syndrome (HARS), may also be accompanied by fat accumulation in the upper back/neck (dorsocervical region) and/or depletion of subcutaneous adipose tissue from the abdomen, face, limbs, or buttocks. HARS is estimated to occur in up to 32% of patients and is associated with health risks similar to those of metabolic syndrome. Techniques to detect and measure HARS include physician and patient assessments and radiologic or anthropometric methods.

## Background

Effective antiretroviral therapy has reduced AIDS (acquired immune deficiency syndrome) mortality and dramatically increased longevity to the point that the long-term effects of HIV (human immunodeficiency virus) infection and treatment are manifesting themselves [1,2]. One particularly troublesome condition associated with long-term treatment of HIV/AIDS is an alteration of fat deposits in the body. In the late 1990s, reports of unusual changes in body fat distribution in HIV patients began to appear in the peer-reviewed literature [3-8].

Today, after almost a decade of study, the disturbance of fat metabolism in HIV-infected patients remains inadequately understood and controversial [9-12]. Abnormal accumulations of intra-abdominal fat [4,8,13-15], enlarged dorsocervical fat masses [6,16-18], and fat loss from the arms and legs, face, and buttocks [3,9,19-21] are the most visible signs of metabolic disturbance in HIV-infected patients with this syndrome. The term "lipodystrophy" is broad and is traditionally used to describe the several morphologic changes related to fat distribution, e.g. lipoatrophy (the loss of fat) and lipohypertrophy (fat accumulation). Both lipoatrophy and lipohypertrophy can occur separately or together in an individual.

In some HIV-infected patients, the body habitus changes are characterized by increases in trunk fat, including accumulation of visceral adipose tissue (VAT), which may present as abdominal obesity or (more rarely) dorsocervical fat accumulation ("buffalo hump"). The term HIV-associated adipose redistribution syndrome (HARS) has been used to describe this syndrome [22-27], even though it does not strictly represent "redistribution" of fat from one depot to another. The fat accumulation in HARS patients may be accompanied by other metabolic disturbances including insulin resistance, glucose intolerance, hypertension, and dyslipidemia, as well as by body image distress [24,28-30]. HARS may also be accompanied by lipoatrophy, typically involving loss of subcutaneous fat of the face, arms, legs, and/or buttocks. Although the combination of visceral adiposity and metabolic disturbances is not unique to HIV, the pathogenesis and clinical presentation appear to differ from those of "standard" obesity in the general population.

In this review, we address the following topics regarding central accumulation of fat in HIV-infected patients: HARS characterization and case definition; health risks associated with HARS; prevalence of HARS; signs of HARS and its relationship to highly active antiretroviral therapy (HAART), and HARS clinical presentation.

### Lipohypertrophy and lipoatrophy: changing perspectives

Peripheral and central lipoatrophy affecting subcutaneous fat is often associated with HIV infection [9,12,31]. Although central fat accumulation was noted in early reports [5,7,16], appreciation of the clinical significance of HIV-associated fat accumulation has come slowly in studies of HIV-associated lipodystrophy.

There may be a number of reasons why fat accumulation has received less attention than peripheral lipoatrophy in the literature. Firstly, there appears to be a perception that fat loss is more common than fat accumulation [9,32]. Secondly, patients may report fat loss more readily to their physicians because of the undesirable cosmetic effects of fat loss from the face, buttocks and extremities [33]. Thirdly, the presence of obesity may confound the detection of HIV-associated accumulation of fat [34]. Fourthly, accumulation of VAT is not readily amenable to objective measurement in the clinical setting [35,36], and sophisticated imaging equipment is needed to visualize and specifically quantify HIV-associated accumulation of VAT [36].

The 1998 Advisory Committee of the International Association of Physicians in AIDS Care (IAPAC) estimated that approximately 1/3 of all treated HIV-infected patients show evidence of intra-abdominal (visceral) fat accumulation along with, or independent of, generalized obesity [8]. This observation coincided with the introduction of one of the first protease inhibitor (PI) drugs (indinavir) that may have contributed significantly to lipodystrophy with VAT accumulation [8]. In summary, HARS is a subset of HIV-associated lipodystrophy in which there is abnormal accumulation of trunk fat, including VAT, appearing with or without concurrent lipoatrophy of subcutaneous adipose tissue (SAT) in the limbs and face, or both. Between 5% and 10% of HARS patients exhibit dorsocervical fat accumulation [24].

To date, it has not been possible to bring the diverse observations of altered fat distribution and metabolism under the rubric of a single syndrome [32], and case definition remains a work in progress [37]. Without a clear definition of what constitutes a "case" of HIV lipodystrophy, the field will continue to be plagued by a wide variation in prevalence estimates, uncertainty about risk factors and indecision about proper course of treatment [38].

### HARS case definition

Methodologic difficulties have plagued investigations of HIV lipodystrophy. Differing case definitions, reliance on detection methods with various levels of sensitivity and specificity, and enrollment of different patient populations have made it difficult to compare results across studies. A low level of agreement between clinical and patient assessments of fat redistribution has also contributed to the problem of clarifying the case definition [39]. Finally, the cross-sectional design of many studies is appropriate for generating hypotheses about the possible association of factors in lipodystrophy, but such studies cannot be used to identify causal relationships.

To resolve these ambiguities, the HIV Lipodystrophy Case Definition Study Group developed a statistical model for the diagnosis of lipodystrophy (including age, sex, duration of HIV infection, HIV disease stage, waist-to-hip ratio [WHR], anion gap, serum high-density lipoprotein-cholesterol [HDL-C] concentration, trunk-to-peripheral fat ratio, percentage leg fat, and intra- and extra-abdominal fat ratio as variables) with a quantitative scale for detecting cases [40,41]. The model efficiently captured cases of HIV lipodystrophy (70% sensitivity, 80% specificity), but this approach failed to develop a model specifically for peripheral fat loss or central fat gain.

In fact, this case-definition exercise deliberately excluded patients with moderate or severe "abdominal obesity" without lipoatrophy on the assumption that it was "age-related adiposity", and was thus biased against those with central fat accumulation or HARS. Nevertheless, regional fat accumulation was a prominent feature of the model with high scores for fat accumulation on the dorsocervical spine and abdominal region. There was no difference in body mass index between the cases and the comparison subjects (24.1 ± 3.7 vs 23.8 ± 3.8, P = 0.454). However, in cases of lipodystrophy, WHR was greater (0.95 ± 0.09 vs 0.91 ± 0.07, P < 0.001), intra-abdominal fat was greater (138 ± 88 cm^2 ^versus 97 ± 71 cm^2^, P < 0.001), and the ratio of intra-to-extra-abdominal fat was dramatically greater (3.21 ± 9.87 vs 0.90 ± 1.16, P < 0.001) compared with controls.

### Prevalence of HARS

In the Aquitaine Cohort (1999), in which 61% of the 581 patients were treated with PI, the overall incidence of fat maldistribution was 38% [42]. Some form of peripheral fat loss was observed in 90% of the patients, but 21% had increased abdominal girth and 10% of the patients had both peripheral fat loss and central fat accumulation. At least one metabolic abnormality was seen in 54% of patients; 51% of men and 45% of women had some form of lipid or glucose abnormality. These results led the authors of the Aquitaine study to suggest that there may be 3 separate presentations of lipodystrophy – fat atrophy, fat accumulation, and a mixed syndrome of both fat atrophy and accumulation [42].

A number of studies have consistently reported a significant proportion of patients with central fat accumulation: the Australian Prevalence Survey of Lipodystrophy Syndrome[43]the HIV Outpatient Study (HOPS) [44], the LIPOCO Study [45], the Norwegian patient survey [46], the Swiss cohort study [47], and the Australia cohort survey [43,48,49]. Safrin and Grunfeld (1999) surveyed reports of localized fat accumulation and loss in HIV-infected patients, and offered prevalence estimates of 1% to 56% for fat accumulation, 1% to 24% for regional loss of fat, and 2% to 83% for the pooled lipodystrophy syndromes [10].

Lichtenstein et al. reviewed 15 published surveys with >100 patients with descriptions suggestive of HARS [50]. They determined that prevalence estimates for HARS vary widely, from 9% [51] to 48% [52] (mean 32%), depending on the criteria used for assessment: increased abdominal girth, abdominal lipohypertrophy, abdominal enlargement, central or abdominal fat accumulation, truncal obesity, central fat gain, increased abdominal wall thickness, or pseudo-obesity. From a review of published, large cross-sectional and longitudinal studies, Tien and Grunfeld determined similar estimates [12]; for any lipodystrophy prevalence ranged from 30% to 62%, peripheral lipoatrophy from 22% to 38%, and any central fat accumulation from 18% to 45%. The highest estimates in the ranges reported by Tien and Grunfeld, however, all came from one study, relying on physical examination only and not on anthropometric or other objective measurements [53].

### Fat redistribution and metabolic changes in HIV infection study

The Fat Redistribution and Metabolic Changes in HIV Infection Study (FRAM) was a large cross-sectional analysis of 425 HIV-infected and 152 HIV-negative men, age 33 to 45, in which lipoatrophy or lipohypertrophy were defined as concordance between subject report of the direction of fat change and results of the physical examination [9,31]. The objective of this study was to compare regional fat distribution (determined by self-report, physical examination and magnetic resonance imaging [MRI]) in HIV-positive men and population-based comparison subjects. Whereas previous studies evaluated only the presence of suspected changes, FRAM used bidirectional instruments (increase or decrease, as well as scales) to grade the magnitude of reported change as mild, moderate or severe.

The FRAM study found no correlation between changes in central fat and peripheral fat in HIV-infected men, measured either by self-report or physical examination [9,31]. In fact, the amount of central accumulation of fat was equivalent in HIV-infected men and comparison men without HIV, and peripheral fat loss did not necessarily correlate with central fat loss. The study authors concluded that central fat accumulation and peripheral fat loss are not reciprocal processes; there is probably no "relocation" of fat between these two depots.

The FRAM study results were somewhat controversial, however. The researchers did not control for body mass index and the HIV-negative control patients were significantly heavier than the HIV-infected patients. This may have contributed to the observation that increases in VAT were more common in the control patients [54]. Furthermore, FRAM was a cross-sectional study; longitudinal studies, such as ACTG 5005s (a substudy of ACTG 384), have demonstrated an increase in truncal adiposity with prolonged antiretroviral therapy [55]. In ACTG 5005s, the prevalence of a high WHR increased from 35% at baseline to 47% after 64 weeks of treatment with antiretrovirals. This moderate relative change underscores the importance of knowing baseline values. However, unlike FRAM, the ACTG studies did not include an HIV-negative control group, so the extent to which the WHR compares with age-matched HIV-negative controls is not clear.

The cross-sectional design of FRAM also does not shed light on how quickly VAT might have accumulated after a patient became infected with HIV or after initiating treatment with HAART. Follow-up data from FRAM are awaited with interest. With further study, perhaps the rate of VAT accumulation may help distinguish HARS from VAT accumulation related to general obesity.

### Health risks associated with HARS

Physical symptoms associated with HARS primarily include bodily discomfort due to local accumulation of a large mass of VAT, with abdominal distension, respiratory difficulty (similar to Pickwickian syndrome), umbilical herniation, gastro-esophageal reflux, difficulty swallowing, abdominal cramping, peptic ulcer, and constipation or diarrhea [33]. It remains unknown if or how much the inflammation associated with HIV adds to or enhances the effects of increased VAT.

#### VAT: independent predictor of all cause mortality

Visceral fat has been demonstrated as an independent predictor of all causes of mortality in a recent study of 291 non-HIV-infected men [56]. In the evaluation of all three fat measures (subcutaneous, visceral and liver fat), age and length of follow-up, only visceral fat was a significant predictor of mortality in these non-HIV infected men [56]. The derangements of fat metabolism and fat storage seen in HARS may be similar to those seen in metabolic syndrome [57] which, according to U.S. Cholesterol Education Program Adult Treatment Panel III (ATP III), must include three of the following five criteria: increased waist circumference (>102 cm men, >88 cm women); increased triglycerides (>150 ng/dL); reduced HDL-C (<40 mg/dL men, <50 mg/dL women); high blood pressure (>130/>85 mmHg); and elevated fasting glucose (>110 mg/dL) [58].

This cluster of cardiovascular risk factors associated with metabolic syndrome, and the prothrombotic state associated with them, may predispose HIV-infected patients with HARS to premature cardiovascular disease [59].

### Insulin resistance

VAT is an independent risk factor for insulin resistance [60]. When Goodpaster et al. (1999) examined the effects of weight loss on regional fat distribution and insulin sensitivity in the general population with obesity, they found that the reduction in VAT was the only adipose tissue parameter that predicted the improvement in insulin sensitivity [61].

An estimated 30% to 90% of patients receiving PI agents are insulin resistant, although the incidence of diabetes mellitus is less than 10% [62]. Lipodystrophy (peripheral lipoatrophy and/or lipohypertrophy) is associated with hyperinsulinemia and insulin resistance [13,63,64]. The dorsocervical fat pad or "buffalo hump" has also been associated with insulin resistance in an analysis of two separate studies involving 1765 HIV-infected patients [65]. In addition, impaired glucose tolerance is part of the spectrum of metabolic changes seen in patients taking antiretroviral therapy [29]. The direct effects of PI and nucleoside reverse transcriptase inhibitor agents, and the indirect effects of visceral adiposity and peripheral fat loss, with fatty infiltration of muscle and liver, account in part for the insulin resistance seen in the HIV-infected population [66].

### Cardiovascular risks

Cohort studies suggest that patients with HIV already face an increased risk of acute myocardial infarction (MI) compared with matched non-HIV-infected controls [59,67,68]. In the subset of patients with HARS, the metabolic consequences of fat deposition may further increase cardiovascular risk [59,68,69], although HIV patients tend also to have high rates of other cardiovascular risk factors relative to age-matched HIV-negative individuals, including diabetes, hypertension and dyslipidemia [67]. In addition, protease inhibitor use is a strong predictor of MI in HIV-infected patients [70].

#### Waist-hip ratio and cardiovascular disease risk

WHR is an integrated index of body-composition changes that reflects VAT stores. It is significantly related to cardiovascular disease risk in the general population [71] and strongly predicts both fasting hyperinsulinemia and insulin levels in both HIV-infected and -negative individuals [28,72]. However, the extent to which WHR is a predictor of cardiovascular disease in HIV has not yet been established, especially since the WHR in HIV may be increased by a low hip circumference (from lipoatrophy) in addition to an increased waist circumference.

A recent study investigated cardiovascular disease risk indices in 100 consecutively recruited HIV-infected women and 75 healthy female control subjects [28]. HIV-infected women had more VAT and less extremity fat by computerized tomography (CT) and dual-energy x-ray absorptiometry (DXA) scan and demonstrated a higher WHR than the control population. Among all subjects, WHR, but not HIV status, was significantly related to high levels of C-reactive protein and other cardiovascular disease risk indices. This could suggest that VAT (as indicated by WHR) is accounting for most of the cardiovascular disease risk in both the HIV-positive and HIV-negative cohorts. However, an earlier study found the coronary heart disease risk estimate was greatest in HIV-infected patients who had primary lipoatrophy, compared with those who had either lipohypertrophy or mixed fat redistribution [73].

The ACTG 362 study group found higher-than-normal risk of cardiovascular disease among patients with HAART-induced immune reconstitution, many of whom had lipodystrophy [74]. The rate of atherosclerotic cardiovascular disease was 7.2 events per 1000 person-years (18 of 643 patients). These 18 patients experienced 20 atherosclerotic cardiovascular events (10 myocardial infarctions, 3 symptomatic coronary heart disease). Of 433 subjects who agreed to remain in follow-up for evaluating risks of cardiovascular disease and metabolic syndrome, 21% of the men and 38% of the women had signs of metabolic syndrome, 31% had a WHR 0.95 to 1.00, and 21% had WHR >1.00.

In a multicenter study of 788 HIV patients, Samaras et al. found the prevalence of metabolic syndrome was 14% by International Diabetes Federation criteria and 18% by ATP III [75]. Many of these patients (49%) had at least two features of metabolic syndrome (particularly elevated lipids) but did not meet the waist circumference or WHR cutoff criteria for metabolic syndrome [75]. It may be that the waist circumference and WHR criteria for the International Diabetes Federation and the ATP III definitions of metabolic syndrome are set too high and are too insensitive for maximal detection of cardiovascular and diabetic risk in HIV-infected adults [76]. In various general populations and ethnic groups, the optimal criteria for identifying high risk abdominally obese patients (with excess VAT) remains unclear [77].

#### Systemic steatosis, e.g. hepatic and intramyocellular fat accumulation

Comorbid liver disease is common in patients with HIV, and the presence of fatty liver may be caused and/or exacerbated by concomitant hepatitis virus infection, use of certain antiretroviral drugs, chronic inflammation and metabolic derangements [78].

Fatty liver, as well as VAT, in non-HIV individuals is related to metabolic risk factors such as dyslipidemia, insulin resistance and inflammatory markers [79,80]. Similarly, patients with HIV and dyslipidemia have increased insulin resistance, and greater amounts of intrahepatic fat and VAT compared to HIV patients without dyslipidemia or normal controls even when total body fat mass is comparable [81]. The insulin resistance impairs insulin from suppressing lipolysis of triglycerides in SAT stores leading to increased release of free fatty acids. These could contribute to expanded VAT stores, which in turn, can release free fatty acids into the portal circulation (Table 1). The increase in free fatty acids in the portal circulation could lead to a net retention of lipid in the hepatocytes or steatosis, which in turn could contribute to systemic insulin resistance [81,82]. In addition, free fatty acid oxidation stimulates gluconeogenesis and hepatic glucose output and thus favors insulin resistance [83]. This is consistent with data showing that, in patients with HIV lipodystrophy, the severity of insulin resistance is related to the accumulation of fat in the liver rather than to the accumulation of intra-abdominal fat [84].

**Table 1:** Functions of visceral adipose tissue compared with subcutaneous adipose tissue.

**Visceral Adipose Tissue**	**Subcutaneous Adipose Tissue**
Major predictor of insulin resistance	Preadipocytes have greater differentiation capacity
Associated with metabolic syndrome	Unclear association with metabolic syndrome
Less responsive to adipogenic effects of insulin	
Portal drainage	
May enhance lipolysis of truncal subcutaneous adipose tissue	May replenish visceral adipose tissue
Produces more interleukin-6	Produces leptin
Produces more plasminogen activator-1	
More surface glucocorticoid receptors	
High density of surface androgen receptors, inhibit expression of lipoprotein lipase and fatty acid uptake	Estrogen promotes accumulation
Altered glucose uptake	Less glucose uptake
Enzyme 11-β hydroxysteroid dehydrogenase type 1 (11-β HSD1) converts cortisone to cortisol	Enzyme 11-β HSD1 barely detectable
Associated with impaired skeletal muscle fat oxidation	
Associated with atherosclerosis	May protect against atherosclerosis

Adapted from Freedland ES. Role of a critical visceral adipose tissue threshold (CVATT) in metabolic syndrome: implications for controlling dietary carbohydrates: a review. *Nutr Metab (Lond).* 2004;1(1):12. [13]

Excess systemic free fatty acids can also find their way to other tissues. Luzi et al. found that triglyceride levels within the myocytes of the soleus and tibialis anterior muscles were significantly elevated in HIV-positive patients compared with healthy controls [85]. This increased accumulation of fat in skeletal muscle of patients with HIV and lipodystrophy is associated with insulin resistance; there was an inverse relationship between insulin action and intramyocellular lipid content.

### Psychosocial effects of HARS

Patient-reported health-related quality-of-life issues associated with abnormal fat distribution in HIV-positive patients came to the attention of investigators early [86]. Changes in body shape stigmatize patients [87] and may interfere with treatment adherence [88]. In addition, patients' anxieties regarding long-term skeletal and cardiovascular risks can interfere with treatment decisions [89]. Although it is difficult to isolate the effect of body habitus changes alone on quality of life in HIV patients [90], significant body image distress is associated with adipose tissue maldistribution [30].

### Signs of HARS

Patients with HARS tend to exhibit excess visceral adiposity without increased total body fat, and have more VAT than non-obese HIV-negative people with similar height-adjusted weight [91]. Patients exhibiting signs of HARS also may have a higher WHR, trunk-to-limb fat ratio, and VAT/SAT ratio [36] than would be expected from their body mass index [92]. HIV-infected patients with truncal enlargement had 2.5–4 times more VAT on MRI scan [4] and 4 times more VAT on single-slice CT scan than healthy control subjects of similar age, sex and height. Engelson and colleagues found that VAT volumes could be quite large in HARS patients, and ranged from 2.1–9.8 L in HIV-positive men with truncal obesity compared with 0.3–1.7 L (P < 0.001) in HIV-positive comparison patients without truncal enlargement [4]. Men with truncal enlargement had 6.5 times more VAT than men without truncal enlargement [4].

#### Extra-abdominal fat accumulation

HARS patients may also exhibit fat accumulation at extra-abdominal sites (e.g. "buffalo hump") [6], and patients exhibiting excess dorsocervical fat are likely to have abnormal truncal fat accumulation as well [18,65]. In an analysis of data from two cross-sectional cohort studies of patients with HIV-associated lipodystrophy, all of the patients with "buffalo hump" also reported central fat accumulation, and 97% to 100% reported facial or buttock lipoatrophy [65]. In multivariate analysis, blood pressure and insulin levels were both significantly associated with the presence of a buffalo hump (P ≤ 0.007), as was duration of ritonavir (P = 0.004) or zidovudine (P = 0.005) use. This particular form of fat accumulation is often very troublesome for patients, who may develop sleep difficulties, snoring or sleep apnea [93], as well as limited range of upper extremity and neck motion, neck and back discomfort [93,94]. In addition to buffalo hump, patients may also develop lipomas, which they often find disfiguring [93].

In a cross-sectional analysis in 582 lipodystrophic patients with HIV, the prevalence of suprapubic lipomas was 9.4%, but these lipomas were more common in patients with dorsocervical fat deposition (18.5%), suggesting a common pathogenesis between these two entities [95]. Other risk factors for pubic lipomas were female gender, obesity (body mass index ≥ 30) and shorter duration of HIV infection.

### Detection of HARS

Body measurement of patients with fat redistribution syndrome is fraught with difficulties [96-99]. Studies of HIV lipodystrophy have relied on several different means of detecting and measuring changes in body shape: self-report by means of questionnaires, scales for tabulating clinicians' observations on physical examination, anthropometric formulas, and radiographic techniques. Self-report and questionnaires are open to subjective bias. In general, levels of agreement (tested by kappa statistic) between subjective measures have been poor [39].

Because HARS is marked by depots of VAT rather than SAT, the condition may not always be evident on inspection or physical examination. In patients with mixed lipodystrophy, the decrease in truncal SAT (lipoatrophy) may contribute to a smaller than expected increase in waist circumference or WHR and thus lead to underestimation of the amount of VAT [100]. The 4-year Multicenter AIDS Cohort Study (MACS) suggested that, in at least some HAART recipients, an increase in WHR could be attributed to a relatively lower increase in hip circumference rather than an increased rate of change in waist circumference [101]. Visual inspection on physical exam in some HARS patients may reveal abdominal distension as VAT pushes the abdominal musculature forward [102]. In these, there may be a lack of flabbiness or pinchable subcutaneous fat stretched over a taut abdomen. However, other HARS patients may present with large bellies that are difficult to differentiate from general obesity.

Standard anthropometric tests have the advantage of being readily available to clinicians [103,104]. Anthropometric criteria, based on waist circumference and/or WHR, have been used to estimate the relative mass of visceral fat [24,105,106]. Some studies have applied a composite criterion of waist circumference >88 cm and WHR ≥ 0.95 for men, plus waist circumference >75 and WHR ≥ 0.90 for women to effectively detect HIV-infected patients with high VAT content [24,35,107,108]. These cutoffs for WHR were based on published anthropometric criteria considered to define visceral adiposity and increased cardiovascular risk in adults [106,109]. As mentioned earlier, WHR data may be misleading, especially if a patient has a normal waist but massive loss of fat and muscle from the hips and buttocks. Thus, in order to exclude patients with apparently high WHR due to such dramatically reduced hip circumference, a minimum waist circumference >88.2 cm was set for males and >75.3 cm for females [24]. While these criteria should be subjected to independent validation, it has been suggested that they may be conservative [108].

The most sensitive and specific methods for the detection of VAT are CT scan and MRI because they provide quantifiable data on the location and mass of visceral fat and SAT [110]. DXA is sensitive and produces results within acceptable variability for measuring the subcutaneous fat of the appendages (SAT), but is more difficult for estimation of VAT since changes in SAT and VAT independently affect the mass of truncal fat [111]. Limitations of these methods include: expense and limited availability; radiation exposure associated with CT scan, limit to single-slice and not whole-body studies; few data exist on normal distribution of VAT and SAT, so it is difficult to make comparisons; subcompartments of SAT and VAT are not homogeneous, so whole-body estimates may be simplistic.

Useful as these radiologic techniques are, CT, MRI and DXA instruments are too expensive to be practical for the identification of HARS patients in routine clinical practice. Routine measurement of waist circumference and/or WHR may be helpful and are recommended for all patients as it enhances diagnostic sensitivity for cardiovascular risk factors [112].

## Conclusion

Body habitus changes resulting from either lipoatrophy or lipohypertrophy, or both, have been combined under a single syndrome known as lipodystrophy. HARS is a specific form of HIV-associated lipodystrophy characterized by abnormal accumulation of trunk fat, including VAT, and may appear with or without concurrent lipoatrophy of SAT. The best current prevalence estimate for HARS is up to 32% of HIV-infected patients. In addition to disfiguring the patient's appearance (enlarged abdomen, "buffalo hump," perhaps exacerbated by peripheral lipoatrophy), HARS is associated with metabolic disorders and health risk factors similar to those of metabolic syndrome. A more precise definition of HARS will require a consensus statement inclusive of clinical manifestations and symptoms. Further research in this area is necessary to understand this complex condition.

## Abbreviations

ACTG AIDS Clinical Trials Group

AIDS Acquired immunodeficiency syndrome

ATP III U.S. Cholesterol Education Program Third Adult Treatment Panel

CT Computerized tomography

DXA Dual-energy x-ray absorptiometry

FRAM Fat Redistribution and Metabolic Changes in HIV Infection

HAART Highly active antiretroviral therapy

HARS HIV-associated adipose redistribution syndrome

HDL-C High density lipoprotein cholesterol

HIV Human immunodeficiency virus

HOPS HIV Outpatient Study

IAPAC International Association of Physicians in AIDS Care

MACS Multicenter AIDS Cohort Study

MI Myocardial infarction

MRI Magnetic resonance imaging

PI Protease inhibitor

SAT Subcutaneous adipose tissue

VAT Visceral adipose tissue

WHR Waist:hip ratio

## Competing interests

Dr. Eric Freedland is currently an employee of EMD Serono, Inc. EMD Serono, Inc. holds rights to Serostim^® ^[somatropin (rDNA origin) for injection], a brand of recombinant human growth hormone, which has been submitted to the FDA for approval for the treatment of HARS. Dr. Balasubramanyam and Dr. Sekhar have received honoraria for consultative meetings with EMD Serono regarding the possible use of growth hormone to treat HIV lipodystrophy. The present manuscript does not discuss growth hormone treatment of HIV lipodystrophy in this condition. Dr. Lichtenstein has nothing to disclose.

## Authors' contributions

All authors were involved in drafting the manuscript and provided extensive comments and review. All authors performed analysis and interpretation of data. All authors have read and approved the final manuscript.
